# Gene Ontology consistent protein function prediction: the FALCON algorithm applied to six eukaryotic genomes

**DOI:** 10.1186/1748-7188-8-10

**Published:** 2013-03-27

**Authors:** Yiannis AI Kourmpetis, Aalt DJ van Dijk, Cajo JF ter Braak

**Affiliations:** 1 Biometris, Wageningen University and Research Centre, 6700AC Wageningen, The Netherlands; 2 Applied Bioinformatics, Plant Research International, Wageningen University and Research Centre, 6700AC Wageningen, The Netherlands; 3 Current address: Functional Genomics, Nestlé Institute of Health Sciences, Campus EPFL, Quartier de l’Innovation, 1015 Lausanne, Switzerland

**Keywords:** Protein function prediction, Gene Ontology, Evolutionary optimization

## Abstract

Gene Ontology (GO) is a hierarchical vocabulary for the description of biological functions and locations, often employed by computational methods for protein function prediction. Due to the structure of GO, function predictions can be self- contradictory. For example, a protein may be predicted to belong to a detailed functional class, but not in a broader class that, due to the vocabulary structure, includes the predicted one.

We present a novel discrete optimization algorithm called Functional Annotation with Labeling CONsistency (FALCON) that resolves such contradictions. The GO is modeled as a discrete Bayesian Network. For any given input of GO term membership probabilities, the algorithm returns the most probable GO term assignments that are in accordance with the Gene Ontology structure. The optimization is done using the Differential Evolution algorithm. Performance is evaluated on simulated and also real data from *Arabidopsis thaliana* showing improvement compared to related approaches. We finally applied the FALCON algorithm to obtain genome-wide function predictions for six eukaryotic species based on data provided by the CAFA (Critical Assessment of Function Annotation) project.

## Background

Central aim of computational protein function prediction methods is to provide reliable and interpretable results, in order to be useful for the biological community. For this reason, prediction methods often make use of the Gene Ontology (GO) controlled vocabulary [1] to describe functional properties of proteins. GO terms are organized in three separate domains that describe different aspects of gene and protein function: Molecular Function (MF), Biological Process (BP) and Cellular Component (CC). Within each domain the terms are arranged in a Directed Acyclic Graph (DAG). Due to the hierarchical structure of the GO-DAG, a protein that is assigned to a particular term is by definition assigned to all of its predecessors, which are more general GO terms. On the other hand, if a protein does not perform a particular function, it is not assigned to the corresponding GO term, nor to any of the successors (more detailed terms) of that term. This constraint of the GO-DAG is referred to as the True Path Rule (TPR) and provides a framework to ensure that functional descriptions of proteins are not self-contradictory. Computational methods often neglect the TPR in their predictions, making their interpretations problematic. Taking the GO DAG (and thus TPR) into account in protein function prediction may lead to improvement of the performance and interpretation.

Violation of TPR can be described in a continuous or in a discrete manner. In the former, the probability (or confidence) of membership to a GO term does not decrease monotonically when moving from more general GO terms to the more detailed ones. Therefore the space of probability vectors (where a vector denotes the joint set of per-GO term probabilities of memberships) can be divided in two sets: one set (*C*) that contains the probability vectors that satisfy the monotonicity constraint and another set (*V*) that contains those that violate the constraint. The challenge from the continuous point of view is, given a vector V to find an optimal corresponding vector in *C*, according to a criterion.

Obozinski et al. [2] developed different “reconciliation” approaches to infer consistent probability vectors from Support Vector Machines (SVM) outputs transformed to probabilities. Performance comparisons between methods based on Belief Propagation, Kullback-Leibner minimization and Isotonic Regression (IR), showed that the last outperformed the rest. In IR [3,4] predictors are the ranks in the ordering of terms in the GO-DAG from general to detailed and the responses are the membership probabilities. The aim is to identify the probability vector that minimizes the squared error with the original input vector and that is monotonic for the predictors and thus belongs to *C*.

**Figure 1:**
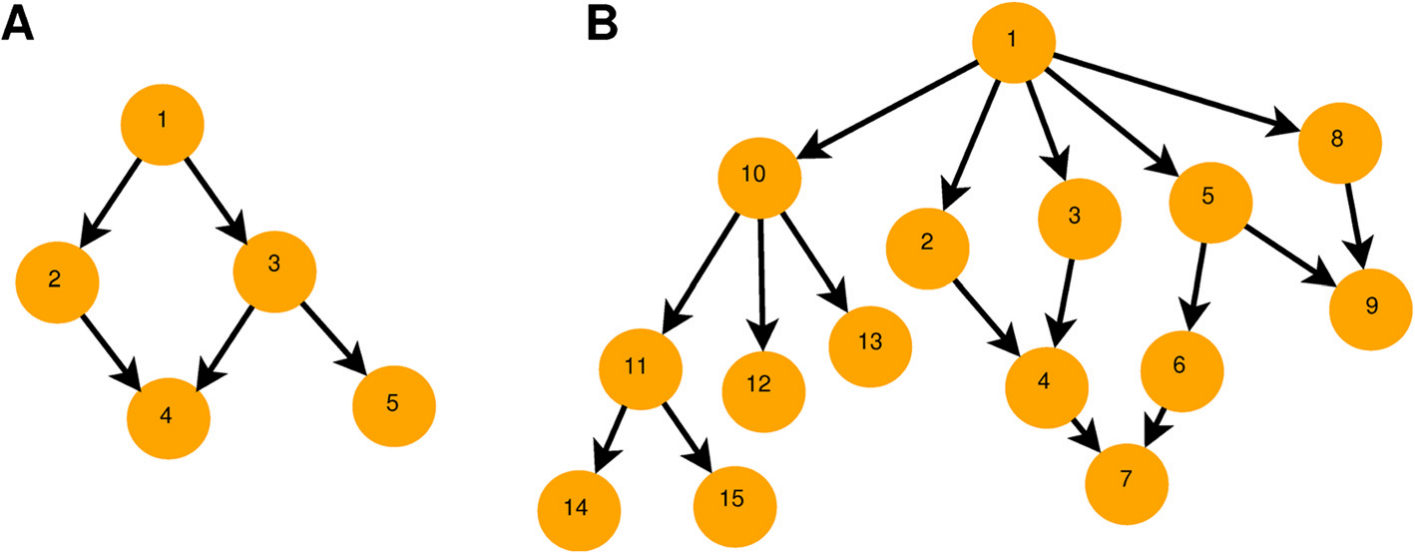
**Example graphs. A** Minimal Directed Acyclic Graph (DAG). **B** DAG with 15 nodes, that was used in our experiments.

In the discrete case, the interest is shifted from the probabilities of membership to the memberships themselves. The TPR violation can be evaluated by checking whether all dependencies are satisfied or not. Given an inconsistent probability vector, the aim is to find the most probable set of GO assignments that do not violate TPR. The task of inferring the most probable latent binary vector given the input probabilities is a decoding problem, which is well-studied in information theory when the underlying structure of constraints has a tree-like structure (including chains). The Viterbi algorithm [5] (also called min-sum [6]) performs such exact inference in tree-like structures. Standard hierarchical classification is not a suitable approach to this problem due to the the DAG structure of GO and multi-functionality of proteins [7]. For instance, applying hierarchical classification to the DAG depicted in Figure 1A, one starts from the root (*x*_1_) and moves to either *x*_2_ or *x*_3_. Regardless the outcome of this classification, it is not possible to give a positive prediction for *x*_4_ without violating the TPR (since exactly one of its parents will not be predicted). However, Vens et al. [8] proposed an hierarchical classification methodology adapted for the GO vocabulary. Other interesting approaches come from fuzzy classification [9]. Exact inference in DAG structures is an NP-hard problem [10] that can be performed by the Junction Tree algorithm [11] but the computational cost is intractable for the size of graphs such as the GO. Barutcuoglu et al. [12] modeled the GO-DAG as a Bayesian Network and they combined SVM outputs per GO term in order to obtain GO assignments. In their case, exact inference was feasible because of the small size of the GO-DAG part used in the study (105 terms). Another related approach was developed by Sokolov and Ben-Hur [13] where SVM classifiers for structured spaces, such as the Gene Ontology, were developed. Valentini et al. [14] and Cesa-Bianchi et al. [15] developed ensemble algorithms that transfer the decisions between base (GO term) classifiers according to the GO DAG structure. Jiang et al. [10] first converted the GO DAG to a tree structure and then applied exact inference.

Here, we take a discrete approach to the problem of TPR violations and we develop an algorithm for the inference of most probable TPR consistent assignments using per-GO term probabilities as input. To the best of our knowledge there is no other algorithm for this task that is suitable for large DAGs. We model the GO DAG as a Bayesian Network and we infer the most probable assignments employing the global optimization method of Differential Evolution [16], which is adapted for discrete space. We test our algorithm on small graphs of size 6 and 15 nodes, for which we can perform exact inference. We show that our algorithm consistently finds the correct optimal configuration. Further we evaluate the performance of the algorithm on probabilistic outputs of Bayesian Markov Random Fields (BMRF) [17] as applied previously in *Arabidopsis thaliana* protein function prediction [18]. Our algorithm is applied to a graph that contains 1024 GO terms. We show that besides providing consistent predictions, our algorithm improves the performance of the predictions compared to a supervised method used in a previous study. We finally applied our algorithm in large scale and we provide function predictions for 32,000 unannotated proteins from six eukaryotic species.

## Materials and methods

The GO is a vocabulary that describes the functions and locations of genes and its terms are arranged in a DAG structure, *i.e.* every node has zero, one or more parents and children. A protein can be assigned to one or multiple terms from each domain of GO [7]. The TPR of the GO-DAG implies that when a protein is known to be assigned to a particular GO term, it should also be assigned to all ancestor terms. In contrast, when a protein is known not to be a member of a GO term, it should not be a member of any of all the successors of that term. By GO-DAG consistency we denote satisfaction of the TPR (also see Table 1). In terms of prediction, given a probably inconsistent vector of input probabilities, one has to find the most probable multiple and consistent GO-DAG paths that the protein has to be annotated to.

**Table 1:** Parent-child relationship in a GO-DAG

		**Parent**	
		1	0
Child	1	C	V
	0	C	C

C denotes consistent configuration while V denotes violating configuration.

Naturally, methods that treat GO terms independently and neglect the DAG structure of the GO can make predictions that are inconsistent. In particular for probabilistic methods those inconsistencies may appear in the form of *p*_*i*_>*p*_*j*_ in which the term j is an ancestor of term i, and thus more general. In this study, we aim to find the most probable consistent GO term assignments, using such probability vectors as input. We first describe the general probabilistic setting, then derive two likelihood based objective functions and finally an evolutionary algorithm for the optimization.

### Bayesian network modelling of GO

Consider a Directed Acyclic Graph (DAG) *G*=(*V*,*E*) with nodes *V* (denoting the set of GO terms) and *E* directed edges (the set of parent-child relationships). Vector **θ** denotes the input probability vector which is |*V*| - dimensional and **x** is the corresponding binary labeling, where *x*_*g*_=1 denotes membership for a particular protein to the *g*-th GO term in *V* GO term.

We model the GO-DAG as a Bayesian Network, with density for **x**:

(1) $$p\left(x|G,\theta \right)=\overset{|V|}{\underset{g=1}{\prod }}p\left(x_{g}|x_{\text{pa}(g)}\right)$$

where *pa*(*g*) denotes the parent set of node *g* and **x**_*pa*(*g*)_ the set of labels that correspond to those parents.

The probability *p*(*x*_*g*_∣**x**_*pa*(*g*)_), under the DAG constraints, is given using the Conditional Probability Table (CPT) of Table 2. The table shows that when *min*(*x*_*pa*(*g*)_)=0 (*i.e.* at least one of the parents has label 0) then *x*_*g*_=0 with probability 1. Otherwise *x*_*g*_=1 with probability *θ*_*g*_ and *x*_*g*_=0 with probability 1−*θ*_*g*_. Note that all inconsistent labelings have zero probability.

**Table 2:** Conditional probability table, under the DAG constraints

		***min*** (**x**_***pa*(*g***_)**)**	
		1	0
*x*_*g*_	1	*θ*_*g*_	0
	0	1 - *θ*_*g*_	1

Given equation 1 and conditional probability tables with parameters = (*θ*_1_,⋯,*θ*_∣*V*∣_), one wishes to identify the most probable labelings vector *x*. There are two challenges in this. The first is how to choose the parameter vector **θ**, discussed in this section, and the second is how to search for the most probable labelings vector *x*, which is discussed in the next section.

Most computational methods for GO term prediction are developed under a multi-class classification framework, where each GO term denotes a class and for each protein the probability of being member of that class is evaluated by the method. Classes are arranged according to a DAG hierarchy and further each protein may belong to one or multiple classes. In GOStruct [13] a SVM approach was developed to perform multi-class classification in a single step. However, the vast majority of the methods split the multi-class problem in multiple binary classification ones (*i.e.* one versus all) and therefore act per GO term and disregard the GO hierarchy. GeneMANIA [19], Kernel Logistic Regression [20] and BMRF [17] propagate function information through networks of protein associations and this operation is performed per GO term. Blast2GO [21], GO-At [22] and Aranet [23] perform overrepresentation analysis for each GO term separately. Such methods do not return the conditional probabilities in the sense of equation 1. The membership probabilities that they return are perhaps best viewed as marginal probabilities, *i.e.* summed over all configurations for GO terms other than the specific term *g*. We might have tried to retrieve *θ* from the relation between marginal and conditional probabilities, but this is certainly not an easy way. We attempted other ways.

Methods such as BMRF return low probabilities for detailed GO terms and high ones for general terms. Prioritization of the proteins in a particular GO term can then be achieved by simply sorting them. By contrast, prioritization of GO terms for a particular protein (a more important task) is not simple as the sets of probabilities for different GO terms are not directly comparable. To make them comparable, the probabilities need to be calibrated. We derive two approaches.

The first, called DeltaL is based on the maximization of the difference of the likelihood and prior probability of the labelings as they are defined in equation (1). The second, called LogitR, is based on explicit calibration of the input probability vector.

For DeltaL, we modify the objective function of equation (1) by incorporating the prior probabilities of membership *θ*^∗^. The prior probability $\theta_{g}^{*}$ depends on the generality of the GO term *g* (*i.e.* the class size) and is estimated as the fraction of the total proteins annotated to that GO term. We use the log ratio between the input probability and the prior, $\text{log}(p(x_{g}|\theta_{g})/p(x_{g}|\theta_{g}^{*}))$ as score function for the labeling of the *g*-th GO term. For *x*_*g*_=1 the score is equal to $\text{log}(\theta_{g}/\theta_{g}^{*})$, while for *x*_*g*_=0 it takes the value of $\text{log}((1-\theta_{g})/(1-\theta_{g}^{*}))$. When $\theta_{g}>\theta_{g}^{*}$ then *x*_*g*_=1 maximizes the function. In the opposite case *x*_*g*_=0 gives the maximum. The extended function is given by the difference of the log likelihoods:

(2) $$\mathrm{\Delta L}\left(X;\theta ,\theta^{*}\right)=\overset{|V|}{\underset{g=1}{\sum }}\text{log}\frac{p\left(x_{g}|\theta_{g}\right)}{p\left(x_{g}|\theta_{g}^{*}\right)},$$

giving

(3) $$\mathrm{\Delta L}\left(X;\theta ,\theta^{*}\right)=\text{log}\left(p(X|\theta )\right)-\text{log}\left(p\left(X|\theta^{*}\right)\right).$$

Note that when the input probabilities are very close to the priors, the objective function of DeltaL becomes multimodal.

**Figure 2:**
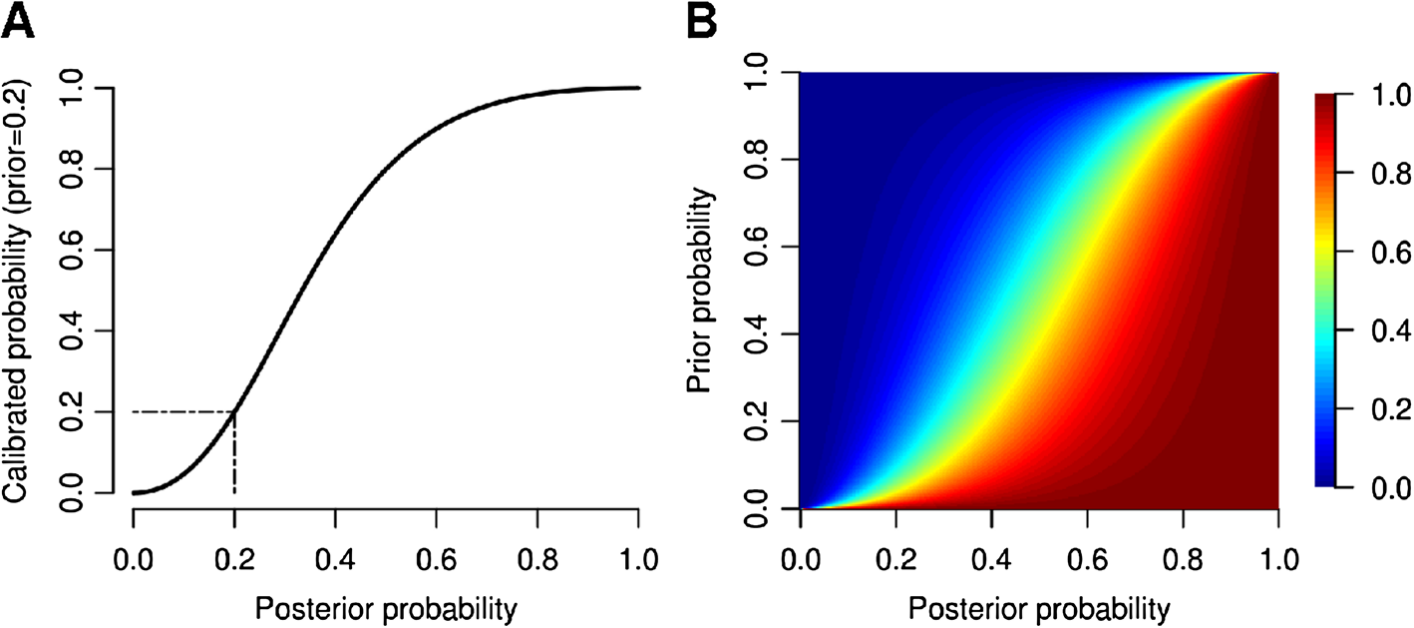
**Calibration of posterior probabilities using *α=2*. A.** Calibrated probabilities (y-axis) against the posterior probabilities (x-axis) when the prior is equal to 0.2. **B.** Image plot, for the entire range of prior and posterior probabilities. The colors denote the calibrated probabilities.

In LogitR optimization of equation 1 is performed on a calibrated input probability vector. The calibration is done as follows:

(4) $$\text{logit}\left(\theta_{\text{cg}}\right)=\text{logit}\left(\theta_{g}^{*}\right)+\alpha \left(\text{logit}\left(\theta_{g}\right)-\text{logit}\left(\theta_{g}^{*}\right)\right)$$

where *θ*_*cg*_ is the calibrated probability for node *g* and can be calculated using the inverse of the logistic transformation, $\theta_{g}^{*}$ is the prior probability of membership for node *g* and *α* a slope parameter. In this objective function, when the posterior probability *θ*=*θ*^∗^ then the probability of membership is equal to *θ*^∗^ (Figure 2A). As *θ* deviates from the prior, the calibrated probability *θ*_*cg*_ changes according to the logistic function given *θ*_*g*_ and *α* (Figure 2B). The *α* parameter was tuned using *Saccharomyces cerevisiae* data. In particular, for a range of *α*=1,1.5,2.0,2.5,3.0 the LogitR approach was applied taking as input BMRF based predictions obtained from a previous study [17] before March 2010. The evaluation set consisted of 327 proteins that were annotated after March 2010, according to the GO annotation file of July 2011. The relevant part of GO DAG contained 423 terms from Biological Process. For each value of *α* the prediction performance was measured using the F-score, which is the harmonic mean of precision and recall. The largest F-score was obtained for *α*=2 and therefore we fixed *α* to that value.

### Optimization by differential evolution: The FALCON algorithm

The DeltaL in equation(3) and LogitR in equation(4) approaches do not involve directly the TPR constraints. We develop an optimization algorithm inspired from Differential Evolution (DE) [16] that by construction is restricted to the subspace of consistent labelings. We call our algorithm Functional Annotation with Labeling CONsistency (FALCON). In general, DE works by evolving a population of candidate solutions to explore the search space and retrieve the maximum. Because DE is derivative free, it has appealing global optimization properties. Also, it is suitable for optimization in discrete spaces (like the labelings space in our problem).

**Figure 3:**
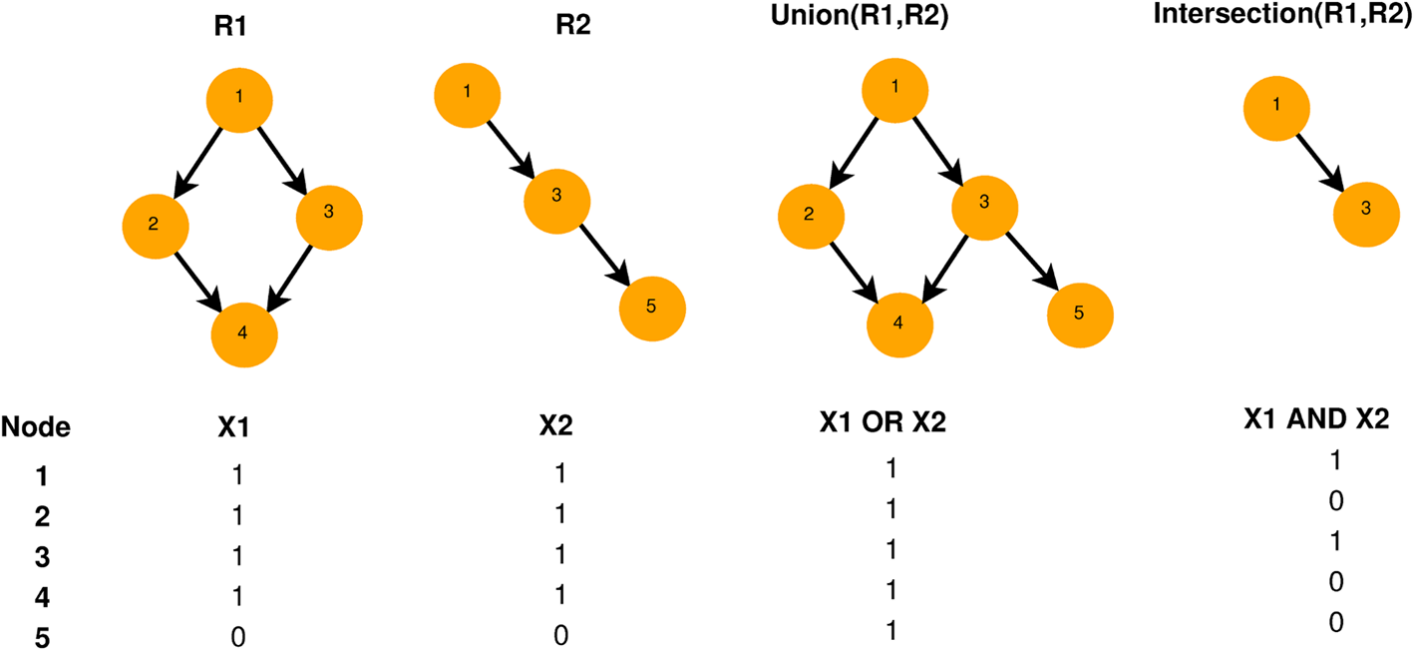
**Examples of graph (upper row) and logical (lower row) operations, using the DAG structure of Figure **1A.

The graph representation of the labelings is helpful to explain how the algorithm works. Given the graph *G* and its corresponding labeling *X*, we define a reduced graph $R=(V_{R},E_{R})$ which contains the nodes with corresponding labels *x*=1. If *X* is consistent, in the TPR sense, R will be a connected sub-graph of *G* and maintaining the original structure for the $V_{R}$ nodes. Consider two labelings *L*_1_, *L*_2_ and their graphs $R_{1}$, $R_{2}$ respectively is given in Figure 3. Graph union $R_{1}\cup R_{2}$ gives the expanded graph $R^{\text{union}}=(V_{R_{1}}\cup V_{R_{2}},E_{R_{1}}\cup E_{R_{2}})$, while graph intersection $R_{1}\cap R_{2}$, gives the contracted one $R^{\text{int}}=(V_{R_{1}}\cap V_{R_{2}},E_{R_{1}}\cap E_{R_{2}})$. The nodes that will be included in the resulting graph are given by set operations (*i.e.*$\left(V_{R_{1}}\cup V_{R_{2}}\right)$ and $\left(V_{R_{1}}\cap V_{R_{2}}\right)$ respectively), but also equivalently by performing logical OR (for union), *X*_1_∨*X*_2_, and logical AND (for intersection), *X*_1_∧*X*_2_ operations on the labelings directly. Table 3 and Figure 3 illustrate those operations.

**Table 3:** Logical operations OR and AND for all the combinations of labels

***x***_**1**_	***x***_**2**_	**OR (*x***_**1**_**∨*x***_**2**_**)**	**AND (*x***_**1**_**∧*x***_**2**_**)**
0	0	0	0
1	0	1	0
0	1	1	0
1	1	1	1

In this example *x*_1_ and *x*_2_ are univariate.

Operations between consistent graphs (labelings) result in consistent graphs (labelings) as well, because the edge set of the last is the union or the intersection of the operands and therefore a particular edge has to pre-exist in at least one of the operands without violating the TPR. This property can be seen as follows: For any parent-child pair of nodes there are three types of configurations that are consistent (Table 1). Graph union and intersection between any combination of those pairs leads to locally consistent labeling. This holds for all the parent-child pairs, so it holds for the full labeling. Therefore the outcome of graph union and intersection will be consistent as well. Further, operations between more than two labelings will be consistent as well due to the associativity property. The FALCON optimization algorithm is based on the generation and evolution of a population of subgraphs *i.e.*$R_{1}\mathrm{...}R_{N}$, with *N*=2∣*V*∣. The population is first initialized with consistent labelings (graphs) and evolved exploiting the graph-union and graph-intersection operations between individuals. Through the generations, all the constructed labelings will be consistent due to the abovementioned property. In our optimization problem we used four strategies to propose a new candidate solution (labelings) for the *i*-th graph $R_{i}$, that is member of the population: 

• S1: Global Union $R_{\text{Cand}}=R_{1}\vee R_{2}\vee e$

• S2: Global Intersection $R_{\text{Cand}}=R_{1}\wedge R_{2}\vee e$

• S3: Local Union $R_{\text{Cand}}=R_{i}\vee R_{1}\vee R_{2}\vee e$

• S4: Local Intersection $R_{\text{Cand}}=R_{i}\wedge R_{1}\vee R_{2}\vee e$

The first two types are called global because they do not involve $R_{i}$ while the latter two are local moves. Graph *e* is a random subgraph of the original full graph (*i.e.* GO-DAG), constructed by sampling a random node and all its ancestors. *e* ensures that all consistent configurations can be eventually proposed and reached.

With *f* the objective function *i.e.* being DeltaL or LogitR, the scheme of the FALCON algorithm is as follows: **Initialize** Population R of size *N*=2∣*V*∣ by picking random consistent vectors (see below): **while** Convergence or Maximum generations not reached **dofor***i*=1 to *N***do** Sample two labelings from the population $R_{1},R_{2}\neq R_{i}$ Construct $R_{\text{Cand}}$ using the a randomly picked strategy *S*1,*S*2,*S*3,*S*4 if $f(R_{\text{Cand}})>f(R_{i})$ then, $R_{i}:=R_{\text{Cand}}$**endforendwhile**

Initialization of the population for DeltaL is done by random sampling GO terms according to their individual score (log ratio of the input and prior probability), while LogitR by sampling from the binomial distribution with probability equal to the calibrated one. In both cases the nodes were up-propagated in order to construct a consistent labeling. The computation was terminated after 10,000 generations or after reaching a plateau (*i.e.* there is no improvement in the objective function for 100 generations). Finally we point that a valid Markov Chain Monte Carlo algorithm cannot be derived using those proposal strategies because they do not represent reversible moves. The bitwise exclusive OR move proposed by Sterns in [24] is reversible but does not lead to consistent labelings. Implementation of the algorithm was done in R language for Statistical Computing and using the igraph R package [25].

### Performance evaluation

We evaluated the performance of the FALCON algorithm on the DeltaL and LogitR objective function using Precision, Recall and F-score. Precision is defined as the percentage of correct GO terms in the list of the GO predictions. Recall is equal to the percentage of the GO assignments that were identified and F-score is the geometric mean of the Precision and Recall.

#### Simulated data

First, we tested the capability of FALCON to retrieve the most probable graph using the full graphs in Figure 1 with hundred simulated probability vectors. The first contains six nodes and the second fifteen. Because the graphs are small, exhaustive search of the most probable labeling was computationally tractable. We generated a hundred random probability vectors, by sampling probabilities for each node from the uniform distribution. Then we identified the most probable labeling for each simulated probability vector and the one returned by FALCON using equation (1) as objective function. Performance measures were calculated by comparing the vectors obtained by FALCON with the most probable ones as calculated from the exhaustive search.

#### Real data

The performance of FALCON was further evaluated using as input the GO membership probabilities of the Arabidopsis proteins as computed by BMRF in [18]. This method provides membership probabilities per GO term independently. We constructed two evaluation datasets from those data. First, we randomly picked 100 Arabidopsis proteins that were already annotated at the time of computing the BMRF posterior probabilities. One constraint was that they should have at least fifty annotations (after up-propagating their original annotations). In this way we ensured that they were annotated in rather detailed GO terms, and therefore the attempt to get GO-DAG consistent predictions would be sensible. Although these proteins had a fixed labeling in the computations, BMRF can calculate membership probabilities for them, by reconstitution, *i.e.* as if they were unknown. The second dataset consisted of 387 proteins that were annotated later than the date of the BMRF computations. Thus, at the time of the computation the proteins were not annotated. We used this second set of proteins to evaluate the performance of FALCON in realistic conditions. In addition, we obtained a further list of predictions using the supervised approach proposed in [18]. In this approach, from the posterior probabilities of the annotated proteins, an F-score based optimal threshold was calculated per GO term. Using this approach, called maxF, we derived a set of predictions for each evaluation dataset. Note that those lists are not guaranteed to be GO-DAG consistent.

## Results and discussion

### Performance of FALCON on simulated data

We initially evaluated the performance of FALCON in the two small graphs of Figure 1. For each graph we simulated 100 probability vectors by drawing from the uniform. Because the graphs are small we could identify the most probable labeling by exhaustive searching. Using equation 1 as objective function and setting all the prior probabilities to 0.5, LogitR retrieved the 98/100 of the labelings for the 6-node graph and 92/100 of the labelings for the 15-node graph. The DeltaL approach also retrieved 98/100 labelings for the small graph (using priors = 0.5 for all the nodes).

### Performance of FALCON on real data

**Figure 4:**
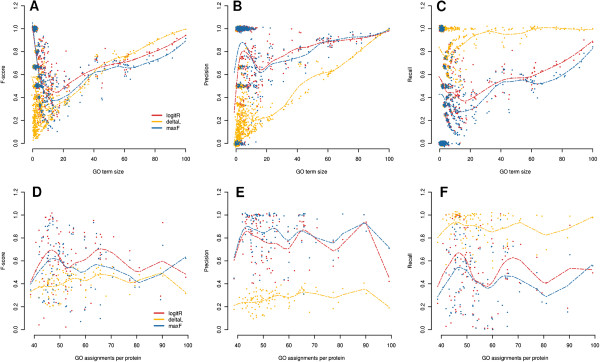
**Performance on the evaluation dataset for the methods LogitR (red), DeltaL (blue), maxF (yellow).ABC.** F-score, Precision and Recall scores for different size of GO terms. **DEF.** The same scores against the number of annotations per protein. Smoothed splines in each subplot show fitted generalized additive models and using the R function smoothṡpline. Because a large number of points in the scatterplot coincided, we performed jittering by adding a small error term to each value *e*∼*N*(0,10^−4^), in order to make the maximum number of points visible.

To assess performance we used Arabidopsis proteins for which we previously calculated GO membership probabilities [18]. The true labelings of the proteins included in the evaluation datasets were known, so we were able to calculate performance metrics. Table 4 shows mean performance measures per protein and per GO term. The LogitR approach leads to the highest F-score, while maxF comes second and DeltaL comes last. We see that all three of them follow the precision-recall trade off (*i.e.* for larger precision there is lower recall and vice versa) with maxF being more precise but with reduced recall and the opposite for DeltaL. LogitR stays in the middle. In Figure 4 performance measures are shown in relation to the GO term level of detail and to the number of GO assignments per protein. Using the F-score to summarize the performance (Figure 4A) we see that for the GO terms that are rather general DeltaL (yellow) performs well, but for the more detailed ones its performance deteriorates. On the other hand LogitR and maxF perform well in detailed GO terms. In terms of Precision (Figure 4B) and Recall (Figure 4C), the latter methods have similar performance but LogitR performs slightly better. On the other hand DeltaL predicts large numbers of terms and therefore shows high recall but low precision, in particular for the detailed GO terms that are of real interest. Comparing the performance of predicting the assignments per protein (Figure 4D-F), the LogitR approach performs consistently better than the others in terms of the proteins that need either small or large number of GO terms to be functionally described.

**Table 4:** Mean performance measures for the evaluation dataset consisting of 100 Arabidopsis proteins

	**LogitR**	**DeltaL**	**maxF**
Per Protein			
Precision	0.79	0.27	0.85
Recall	0.55	0.90	0.46
F-score	0.63	0.41	0.56
Per GO term			
Precision	0.70	0.25	0.81
Recall	0.50	0.80	0.40
F-score	0.70	0.44	0.66

We further evaluated the performance of our approaches using a set of proteins that were annotated after obtaining the BMRF predictions (Table 5). From the total of 387 newly annotated proteins, maxF returned predictions for 84 of them, DeltaL for 328 proteins and LogitR for 147 proteins. Again, maxF and DeltaL show comparable performance while logitL returned an improved list in terms of F score. Further, the higher recall rates of DeltaL tend to give longer lists of predictions. Importantly however, DeltaL and LogitR return predictions that are consistent with GO-DAG and are therefore preferred because such predictions are biologically interpretable.

**Table 5:** Mean performance measures for the newly annotated proteins

	**Precision**	**Recall**	**Fscore**	**Proteins**
maxF	0.34	0.35	0.23	84
DeltaL	0.08	0.58	0.19	328
LogitR	0.26	0.50	0.27	147

Every method predicted different numbers of proteins. The number of proteins returned (out of the total 387) are given in the last column of the table.

### Novel predictions

We performed protein function predictions using the FALCON algorithm on the unannotated parts of the genomes of 6 eukaryotes (human, mouse, rat, slime mold, frog and arabidopsis). This dataset includes the eukaryotic targets used in the Critical Assessment of protein Function Annotation (CAFA) experiment of 2011 [26] and consists of 32,201 proteins. Function predictions were made for 1,917 GO terms from the Biological Process and Molecular Function compartments of Gene Ontology. The input probabilities were computed during CAFA’11 by BMRF integrating protein networks constructed from the STRING database [27] with orthology information obtained from ProgMap [28]. The BMRF and FALCON predictions are available in the BMRF website: http://www.ab.wur.nl/bmrf_yk/FALCON_CAFA.tab.gz.

## Conclusions

Overall, we examined the performance of FALCON for two objective functions, but FALCON is in principle suitable for optimization of a wide range of objective functions. The main purpose of FALCON is to provide GO DAG consistent predictions. We showed that this comes with no loss of the prediction performance. In fact LogitR outperforms the maxF method. The predictions of FALCON are GO-DAG consistent and therefore biologically much easier interpreted by the curators of protein function annotations. In this study an estimate of the calibration parameter *α* for LogitR was obtained using a yeast data set and the input probabilities were obtained from a semi-supervised method (BMRF), but, thereafter, FALCON is unsupervised; it infers the optimal GO term assignment using only the input probability vectors and the prior probabilities per GO term (computed from a set of predictions or using external Gene Ontology information). In contrast, in maxF, a training set is necessary in order to obtain the optimal cutoffs per GO term. In this study both approaches were applicable but the FALCON algorithm is expected to have broader applicability.

## Competing interests

The authors declare that they have no competing interests.

## Authors’ contributions

YK and CtB conceived the study and developed the algorithm. YK and ADJvD performed the BMRF analysis on the CAFA dataset. YK performed the analysis on FALCON and wrote the manuscript. CtB supervised the research and helped in writing the manuscript. All authors read and approved the final manuscript.
